# Robust Classification of Tea Based on Multi-Channel LED-Induced Fluorescence and a Convolutional Neural Network

**DOI:** 10.3390/s19214687

**Published:** 2019-10-28

**Authors:** Hongze Lin, Zejian Li, Huajin Lu, Shujuan Sun, Fengnong Chen, Kaihua Wei, Dazhou Ming

**Affiliations:** 1 School of Automation, Hangzhou Dianzi University, Hangzhou 310018, China; fnchen@hdu.edu.cn (F.C.); 17196220@hdu.edu.cn (D.M.); 2 Alibaba-Zhejiang University Joint Institute of Frontier Technologies, Zhejiang University, Hangzhou 310027, China; zejianlee@zju.edu.cn; 3 Zhejiang Key Laboratory of Design and Intelligence and Digital Creativity, College of Computer Science and Technology, Zhejiang University, Hangzhou 310027, China; 4 Southern Zhejiang key Laboratory of Crop Breeding, Wenzhou Academy of Agricultural Sciences, Wenzhou 325006, China; luhjzw@163.com; 5 Wenzhou Specialty Station, Wenzhou 325006, China

**Keywords:** tea, classification, fluorescence, LED, convolutional neural network, variety, EEM

## Abstract

A multi-channel light emitting diode (LED)-induced fluorescence system combined with a convolutional neural network (CNN) analytical method was proposed to classify the varieties of tea leaves. The fluorescence system was developed employing seven LEDs with spectra ranging from ultra-violet (UV) to blue as excitation light sources. The LEDs were lit up sequentially to induce a respective fluorescence spectrum, and their ability to excite fluorescence from components in tea leaves were investigated. All the spectral data were merged together to form a two-dimensional matrix and processed by a CNN model, which is famous for its strong ability in pattern recognition. Principal component analysis combined with k-nearest-neighbor classification was also employed as a baseline for comparison. Six grades of green tea, two types of black tea and one kind of white tea were verified. The result proved a significant improvement in accuracy and showed that the proposed system and methodology provides a fast, compact and robust approach for tea classification.

## 1. Introduction

The classification of tea is of great interest, for the sample’s geographical origin and authenticity is highly related to its quality as well as market value. Current assessment is conducted by skillful tasters who judge the grade according to appearance, flavor and aroma of tea infusions. The process is intrinsically subjective and biased and can be easily affected by the physical condition of tasters. Therefore, an alternative objective analytical method is in great demand to provide precise and reliable quality information to consumers and help prevent fraudulent labelling. The optical method has been widely applied in the field of food classification due to its non-destructive and rapid response characteristics. Absorption spectroscopy is an important method for food quality control, classification and authentication; it mainly includes UV–Vis absorption spectroscopy [1,2], near-infrared (NIR) spectroscopy [3] and mid-infrared spectroscopy [4,5] accompanied with chemometrics including partial least square (PLS) and linear discriminant analysis (LDA). Laser-induced breakdown spectroscopy (LIBS) can excite the trace elements contained in samples, and classify the species according to the trace element spectrum [6,7]. The main drawbacks of these methods are the expensive cost of the equipment and tough handling requirement both for extracting the organic compounds from samples as well as operating equipment. The detection of tea grade and type based on machine vision is attractive to the food industry for its convenience in system installation and fast response. Yet, its high requirements on sample positioning, lighting uniformity and focal distance still prevent its practical application [8,9,10].

Application of fluorescence spectroscopy in food analysis is becoming increasingly attractive and has been demonstrated to be capable of classifying wine vinegars [11], fermented dairy products [12], camellia oil [13], cereals flours [14] and meat freshness [15,16] etc. Laser-induced fluorescence (LIF) can excite the characteristic fluorescence of the internal materials of leaves [17], which makes the detection more accurate [18], but there is still room for reduction in laser price and maintenance cost. LED-induced fluorescence is a newly developed technology that uses LEDs instead of laser diodes as excitation sources [19]. Due to its high light intensity, high luminous efficiency, easy operation and long lifetime performances, LED is suitable as an excitation source for fluorescence spectroscopy [20]. Combining pattern recognition algorithms, LED-induced fluorescence spectroscopy plays a role in many recognition and classification systems, and has been applied to fields like fruit maturity detection [21] and tea grades classification [22,23]. Due to the heterogeneous contents in tea leaves that get excited by a single LED among each measurement, the fluorescence spectra may vary in a wide range that fails prediction. To solve this problem, multiple LEDs were employed to generate more characteristics, and different architectures of classification models were applied to reach a high classification accuracy.

A convolutional neural network (CNN, or ConvNet) is a deep, feed-forward artificial neural network that being extensively developed in recent years [24]. The most commonly applied field includes image recognition, video analysis, natural language processing, medical image processing and so on. Its advantage is the strong ability in analyzing 2D data. In this work, we designed an optical system to efficiently get fluorescence spectra of tea leaves by illumination of seven different LEDs one by one. The seven 1D fluorescence spectrum were bonded together to form a figure-like 2D matrix and processed by a CNN. The fluorescence spectra of nine different tea samples with different grades and various kinds excited by various excitation LEDs were studied, and classified by the proposed method. The result was compared with the conventional principal component analysis (PCA) combined with k-Nearest Neighbor (kNN) with different training data set.

## 2. Materials and Methods

### 2.1. Samples

Nine dried and fermented tea samples were investigated in this experiment, including six kinds of green tea, two kinds of black tea and one kind of white tea. The green tea samples consist of a Wuniuzao tea (G-WNZ), Huangshan Maofeng tea (G-HM), Vietnam Green tea (G-V) and three different grades of Xihu Longjing tea (G-LJ1, G-LJ2, G-LJ3). G-LJ1 and G-LJ3 are from the same place of origin, the difference is that G-LJ1 harvested during the Qingming festival, and G-LJ2 harvested during the Guyu festival, which is 15 days later. G-LJ2 is of the same species, but produced 50 km away from the other two, and harvested during the Qingming Festival. The black teas include Keemun black tea (B-KM) and Lipton yellow label tea beverage (B-L). The white tea is called Mancheng white tea (W-MC). G-WNZ, G-HM, G-LJ1, G-LJ2, G-LJ3, B-KM and W-MC were collected from local tea plantation and stored in air-tight packaging. B-L and G-V were made by nature tea leaves with some additives and bought from the supermarket.

### 2.2. Apparatus Design and Procedure

**Figure 1:**
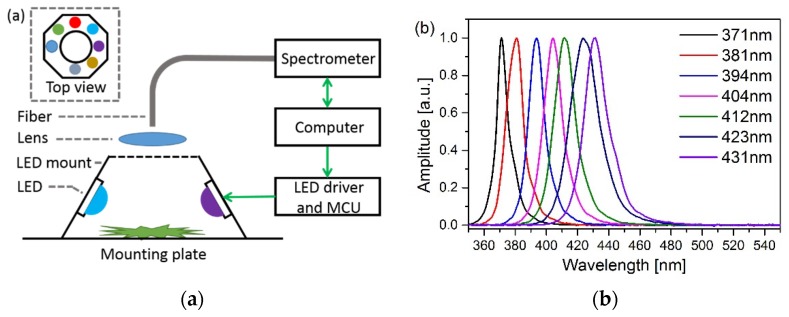
(**a**) Schematic of the multi-channel LED-induced fluorescence system. (**b**) The spectra of the seven excitation LEDs.

Figure 1a shows the schematic of the multi-channel LED-induced fluorescence system. The inset depicts the top view of the LED mount with seven LEDs. From the button to the top, the system was mainly made up of a mounting plate, a LED mount, a lens and a fiber. The mounting plate was made up of black aluminum, so that the reflection of excitation light was minimized. The LED mount, which was created by a 3D-printer, had a top view shape of an octagon which means that up to eight LEDs can be installed. Tea leaves were placed in the middle of the mounting plate, got equidistant illumination from LEDs around with an incident angle of 60°. Their fluorescence was collected with an achromatic lens, focused into a single multimode fused silica fiber with a core diameter of 1.0 mm, and finally transmitted to a spectrometer (FX2000 spectrometer; Fuxiang Inc., Shanghai, China) with spectral range from 200 to 1100 nm and resolution of 0.6 nm. In this experiment, seven LEDs with luminous flux of around 10 lm were employed as excitation lights. Their normalized spectra are shown in Figure 1b, with central wavelengths of 371 nm, 381 nm, 394 nm, 404 nm, 412 nm, 423 nm, and 431 nm, respectively. These seven LEDs were chosen, because their wavelengths can generate the fluorescence of tea efficiently, and their wings of spectra will not exceed 500 nm, where fluorescence would exist. All the LEDs were controlled by a microcontroller unit and synchronized with the spectrometer by a LabVIEW program. The lens and fiber were fixed separately from the plate, preventing drifts that may draw into the optical system when replacing tea leaves.

The procedure of measuring a typical tea sample is depicted below. After placing tea leaves in the middle of the mounting plate, the LEDs were turned on and off sequentially. In 0.2 s after a LED was turned on, the spectrometer started to get the spectrum with an integration time of 1 s, then the LED was turned off and alternate to another one. When all seven LEDs completed, a background spectrum with no LED shining was recorded. Eight rows of spectra were collected for data pre-processing, and each tea were measured for 200 times.

### 2.3. Data Pre-Processing

Pre-processing of seven LED spectrum includes being subtracted by the background spectrum, smoothed with 2nd-order Savitzky–Golay filter and normalized to the maximum amplitude in the 650~700 nm region, where the first peak of chlorophyll exists. Spectra data between 500 nm and 900 nm were remained for analysis, since the data before 500 nm were mainly LED spectra, and those after 900 nm were dominated by noise. After pre-processing, eight rows of data became seven rows (seven different excitation LEDs), each contained 837 elements (fluorescence spectrum at selected wavelengths from 500 to 900 nm). The tea samples were divided into training (50%) and test (50%) sets.

### 2.4. Principal Component Analysis-K-Nearest Neighbors

PCA-kNN is a modification of Principal Component Analysis associated with the K-nearest neighbors model for classification purposes. It is based on the PCA algorithm that searches for latent variable with a maximum covariance for the categorical variables. The new object is then assigned to the kNN method, which will classify unseen objects based on their similarity with samples in the training set. The methods above are implemented through a self-written program based on MATLAB (The MathWorks Inc., Natick, MA, USA).

### 2.5. Convolutional Neural Network

**Figure 2:**
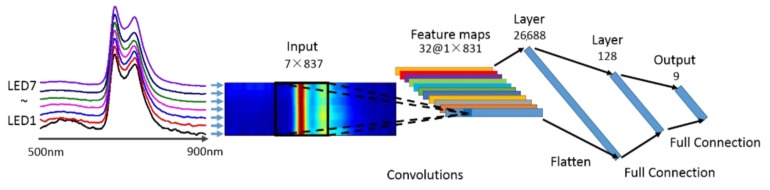
The architecture of our designed CNN.

The architecture of CNN is depicted in Figure 2. The input layer were figures with seven rows and 837 columns, coming from fluorescence spectra of seven LEDs bonded together. The output were nine different tea leave categories. The hidden layers contained a convolution layer, a flattening layer and two full connection layers.

The convolution layer, which is the core building of a CNN, applies a convolution operation to the input. The convolution emulates the response of an individual neuron to visual stimuli. The layer’s parameters consist of a set of learnable filters (or kernels) with small receptive fields, and the size of each filter is 7 × 7. During the forward process, each filter is convolved across the width and height of the input and computes the dot product between the entries of the filter and the input. The convolutional operation finally produces a 2-dimensional feature map of that filter. As a result, the network learns filters that are activated when it detects some specific patterns of the features at some spatial position in the input. In our case, the size of the input figure was 7 × 837, so the filter only slide in the horizontal direction, and the result after one convolution was a vector of size 1 × 831. Finally, 32 kernels were applied to get 32 feature maps.

In the design of a CNN architecture, the pooling operation is a widely-applied method of non-linear down-sampling. The rationale is that the precise location of an activated pattern is less important than its rough location compared with other features. However, in our scenario, a feature is an amplitude relation between a series of exact wavelengths, which means location information is ensured by the spectrometer. Thus, pooling layer was not appropriate and not considered in our network. Instead, a flattened layer was applied to rearrange the feature maps, and then two fully connected layers were used to compute with a matrix multiplication followed by a bias offset, as seen in regular neural networks. In addition, the rectified linear unit (ReLU) [25] was adopted as the non-linear activation operation after each convolutional layer and fully-connected layer. In the output layer, the Softmax operation was used to give the predicted probability. The cross-entropy loss was employed to measure the disagreement between the ground-truth category and the predicted result. To learn the network parameter according to the loss, the stochastic gradient descent (SGD) optimization method with a learning rate of 0.1 without momentum was utilized. The CNN algorithm was performed with Python software and Keras.

## 3. Results

### 3.1. Exploratory Analysis of the Data

**Figure 3:**
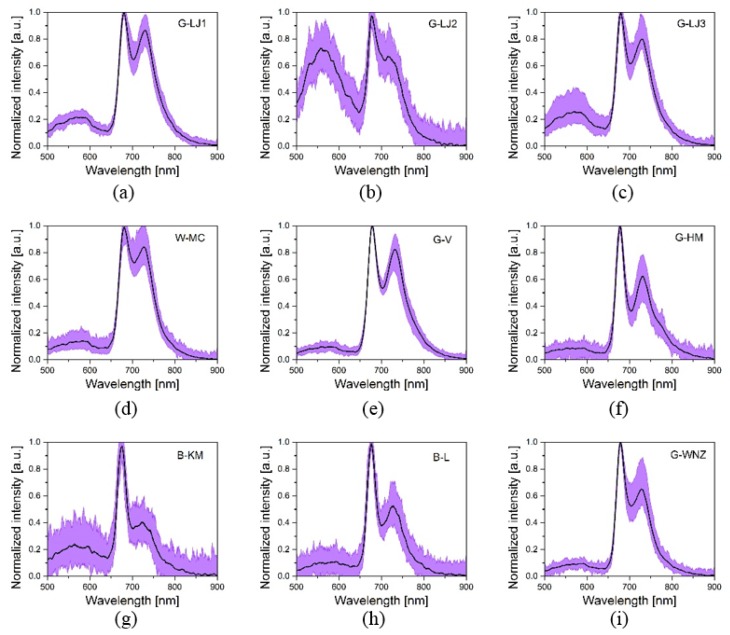
The fluorescence spectra of G-LJ1 (a), G-LJ2 (b), G-LJ3 (c), W-MC (d), G-V (e), G-HM (f), B-KM (g), B-L (h), and G-WNZ (i) under excitation of LED1.

As can be seen in the left part of Figure 2, spectrum of LED1 provides more information than the other six, so LED1 was chosen for exploration in this section. Figure 3 shows the fluorescence spectra of nine kinds of tea under excitation of LED1. The central black line is the average of 100 measurements, and the violet shadow reflects the various among each measurement. Three significant peaks can be observed. The two maxima in the red (near 680 nm) and far-red region (near 740 nm) depend mainly on the concentration of the fluorophore chlorophyll a [26]. The origin of the fluorescence in the wavelength range from 500 to 600 nm is not completely know by researchers at the moment. Possible candidates for this emission under UV-A excitation can be found in the literature (see [27,28] and references in-there). This green-orange emission was often found to be weak with respect to the chlorophyll emission, however, it can be still useful for the discrimination of the different tea kinds by the CNN analysis. The various components provide distinctive responses to selected excitation wavelengths. Thus, tea classes can be discriminated by analyzing the fluorescence spectra.

Comparing the average spectra of each kind, black teas and green teas show an apparent difference in the second peak of chlorophyll around the 730 nm region, where the amplitudes of green teas were all above 0.6, while those of black teas were below. The fluorescence spectra of white tea were similar to those of green teas. This difference may be attributed to the producing process, as green tea is produced without fermentation, and black tea is fully fermented. White tea is 90% fermented, so its fluorescence spectra still share some similarity with those of green tea. Due to high fluctuation, the spectra among green tea or among black tea overlaps largely, and even black tea and green tea may overlap, e.g., G-HM and B-L. Concerning the fluorescence peak in 500–600 nm, the amplitudes of G-LJ1, G-LJ3, B-KM, and especially G-LJ2 were higher than the other tea samples.

### 3.2. PCA Classification with Individual Fluorescence Spectrum

**Figure 4:**
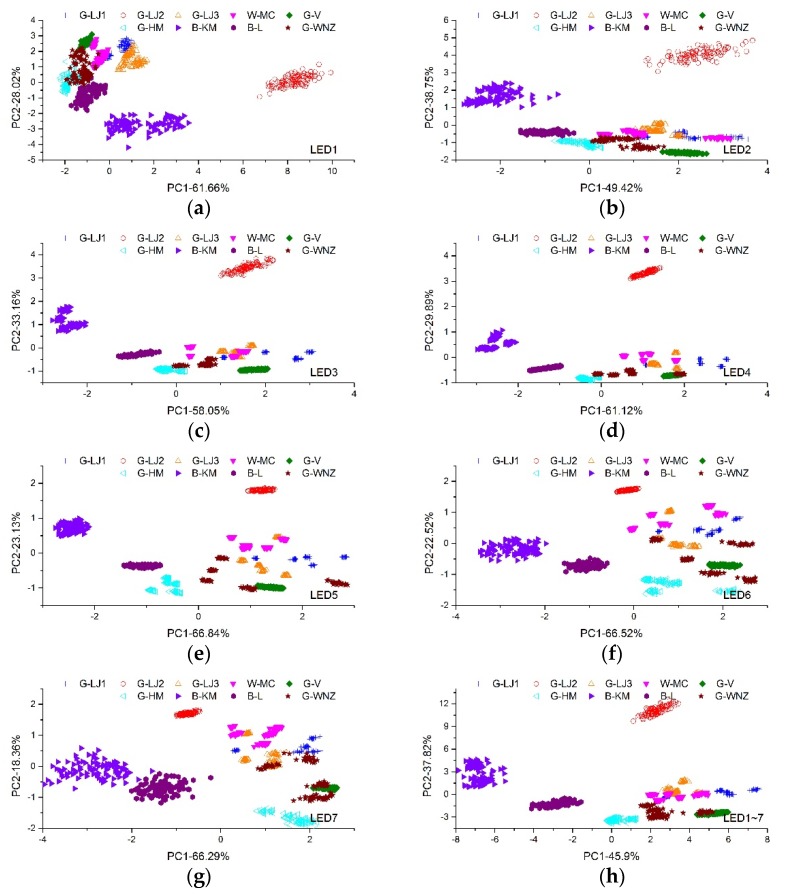
The PCA score plots (PC1×PC2) based on fluorescence spectra of LED1~LED7, respectively (**a**–**g**) and all spectra (**h**).

Figure 4a−g shows the PCA dimension reduction result of the tea samples, according to the fluorescence spectrum excited by LED1 to LED7, respectively. LED1, with excitation wavelength that can generate fluorescence around 550 nm region efficiently, was able to categorize G-LJ2 and B-KM. The other five types of tea overlapped slightly and could not be classified. LED2~LED7 can excite fluorescence of chlorophyll, but has less influence on the emission of other compounds. Thus, the fluorescence spectra of these LEDs were similar, and the result of PCA were alike too. LED2 could classify G-LJ2 and B-KM apart from the other teas, while LED3 and LED4 could distinguish additional B-L. LED5-7 could categorize G-LJ2, B-KM, B-L and G-HM.

PCA combined with the kNN method was also employed to all spectra of seven LEDs. The models were trained in two ways. In one way, all fluorescence spectra of seven LEDs were connected together to form a 1D data, and then processed by the PCA and kNN method. The PCA score plots (PC1×PC2) of this method is shown in Figure 4h. The other way was to extract principal components from individual spectrum firstly and then use all the principal components of seven LEDs for kNN classification.

The results obtained by different models were shown in Table 1. With individual excitation LED, the best accuracy was 0.839 based on the fluorescence spectra induced by LED4. With multiple excitation LED, the proposed two methods showed no significant improvements, reaching accuracies of 0.828 and 0.826, respectively.

### 3.3. CNN Classification with Fluorescence Matrix

**Figure 5:**
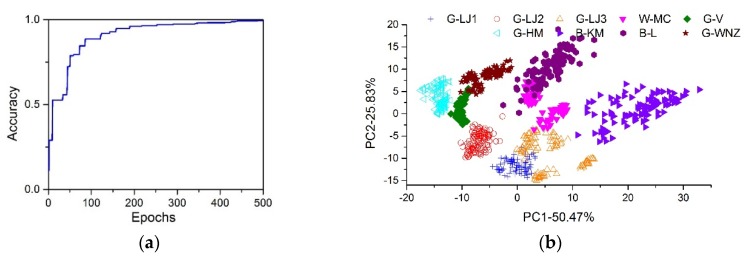
(**a**) CNN prediction accuracy against epochs. (**b**) The PCA score plots (PC1×PC2) based on the outputs of the last but one layer of the CNN.

The neural network was trained for 500 epochs. As can be seen in Figure 5a, the accuracy increased with the expansion of epochs, and the model finally reached an accuracy of 0.993 on the test set samples. The proposed CNN method showed a significant improvement to the classification compared to PCA combined with kNN method, proving its strong ability in feature extraction. To visualize the classification ability of CNN, PCA was utilized to the outputs of the last but one layer of the CNN on test set samples, as shown in Figure 5b. For G-LJ1, G-LJ3, W-MC, G-V, and G-WNZ that overlapped strongly, now they were separated and could be classified with linear classifier e.g., kNN or multiple linear regression. Previous research of CNN applied to the feature extraction of spectrum used the product of 1D spectrum data multiplexing its transposed vector [29]. However, this operation provides no extra data for feature extraction, so no advantages were seen compared to traditional methods. In our case, when applied to the 2D data that truly reflects chemical components inside in a view of figures, CNN can catch the intrinsic relation of fluorescence between different excitation light sources, and thus improves the classification ability.

## 4. Discussion and Conclusions

In this paper, a multi-channel LED-induced fluorescence system combined with a CNN method was proposed. In principle, the system belongs to three-dimensional Excitation–Emission Matrix (EEM) fluorescence spectroscopy, but the system is small, compact and cost-effective compared with the conventional fluorospectrophotometer, or other apparatus employed for tea classification in UV-Vis spectroscopy or FTIR spectroscopy. LED with a central wavelength around 371 nm is proved to be efficient to get fluorescence of not only chlorophyll, but also of other compounds. The second peak of chlorophyll in 720 nm region showed a relationship with tea species. Traditional methods, e.g., PCA combined with kNN, are feasible in tea classification from the individual or multi-channel LED-induced fluorescence spectra in our case, but the accuracy can be improved. The CNN method is more powerful in processing multiple-channel LED-induced fluorescence data. The experimental result reveals that the designed system is a robust tea classification system that can capture and represent the complex correlation between tea leaves of different kinds as well as orientations. In conclusion, the system of multi-channel LED-induced fluorescence system combined with a CNN method can lead to an effective tea varieties recognition. Further studies can be directed to explore the relation between fluorescence spectra and fermentation stage of tea during production.

## Figures and Tables

**Table 1:** The accuracy of the test set samples with models trained by PCA combined with kNN with different training data set. The best performance is highlighted.

Method	PCA+kNN								
Spectra with excitation LED No.	**1**	**2**	**3**	**4**	**5**	**6**	7	1~7	1+…+7
PC number	11	6	3	3	3	4	11	9	41
Accuracy	0.826	0.802	0.796	**0.839**	0.790	0.754	0.828	0.828	0.826
